# Supplementary material for: Preparation of a Series of Pd@UIO-66 by a Double-Solvent Method and Its Catalytic Performance for Toluene Oxidation
Source: Materials (Basel). 2019 Dec 23;13(1):88. doi: 10.3390/ma13010088 (PMC6981644; doi:10.3390/ma13010088)
Supplement: Supplementary file 1 [file materials-13-00088-s001.pdf]

materials

Article

Preparation of a Series of Pd@UIO-66 by a Double-Solvent Method and its Catalytic Performance for Toluene Oxidation

Chuanying Wei, Haili Hou, Ermo Wang and Min Lu *

School of Chemical Engineering, Northeast Electric Power University, Jilin 132000, China; 13844204685@163.com (C.W.); h18704329087@163.com (H.H.); asdfg8596@126.com (E.W.)

* Correspondence:lumin19770919@163.com; Tel.: +86–1357–851–1861

Received: 26 November 2019; Accepted: 20 December 2019; Published: date

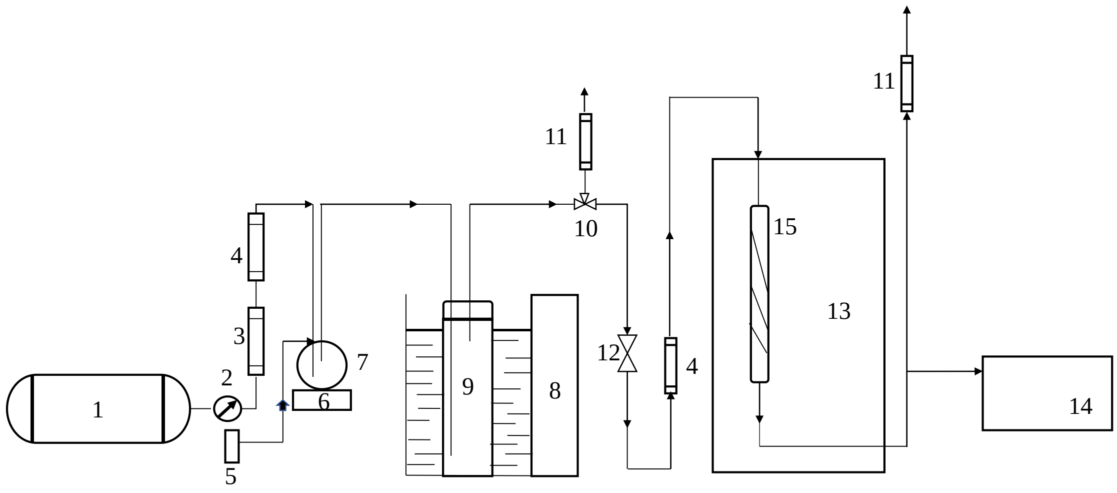

Scheme S1. Schematic diagram of catalytic process: 1. Air compressor 2. Reducing valve 3. Dry pipe 4. Flowmeter 5. Injection pump 6. Hot plate 7. Gas evaporator 8. Thermostatic water tank 9. Buffer bottle 10. Three-way valve 11. Sorbent tube 12. Control valve 13. Reaction generator 14. Gas chromatograph 15. Glass tube.

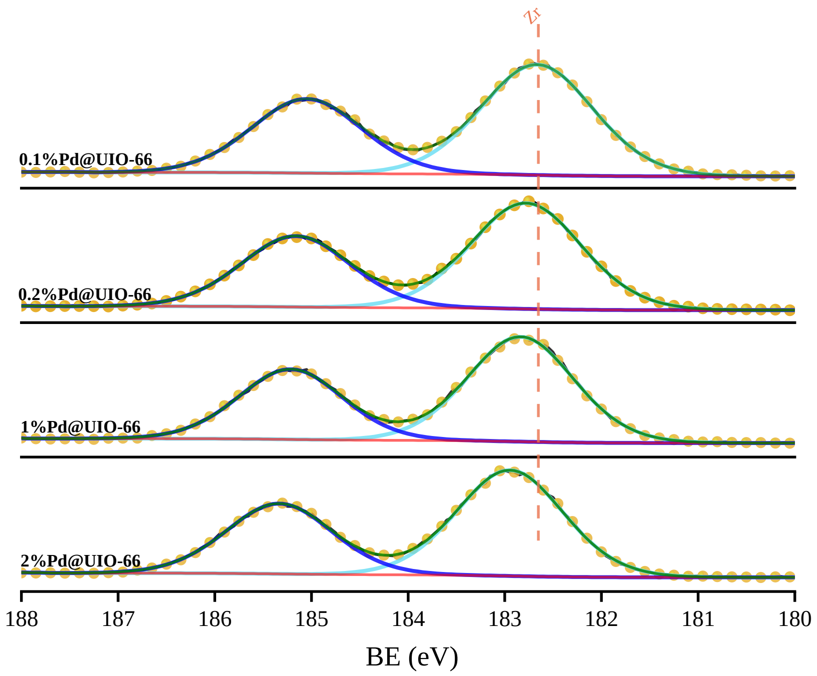

Figure S1. XPS spectra of Zr3d.

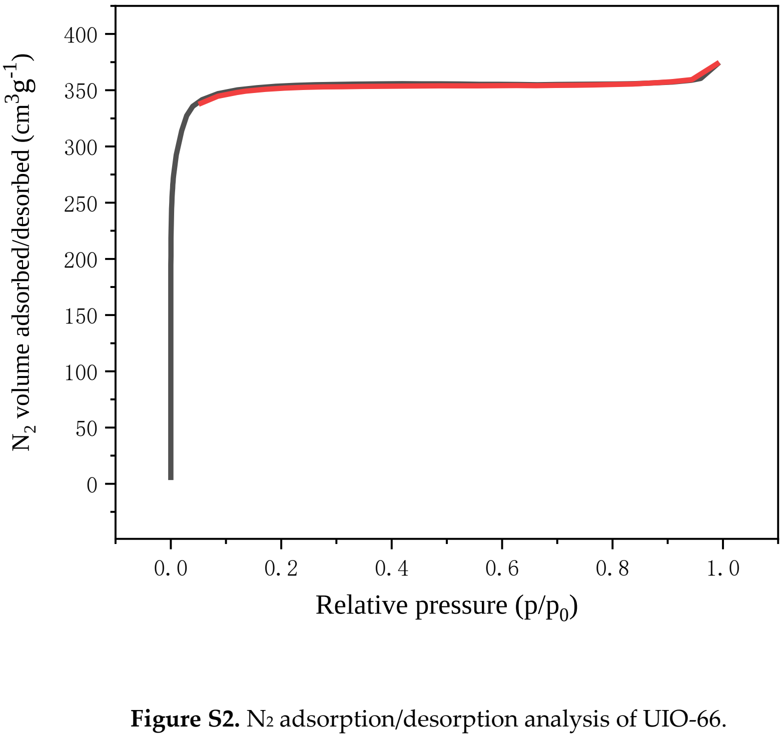

Figure S2. N_2 adsorption/desorption analysis of UIO-66.

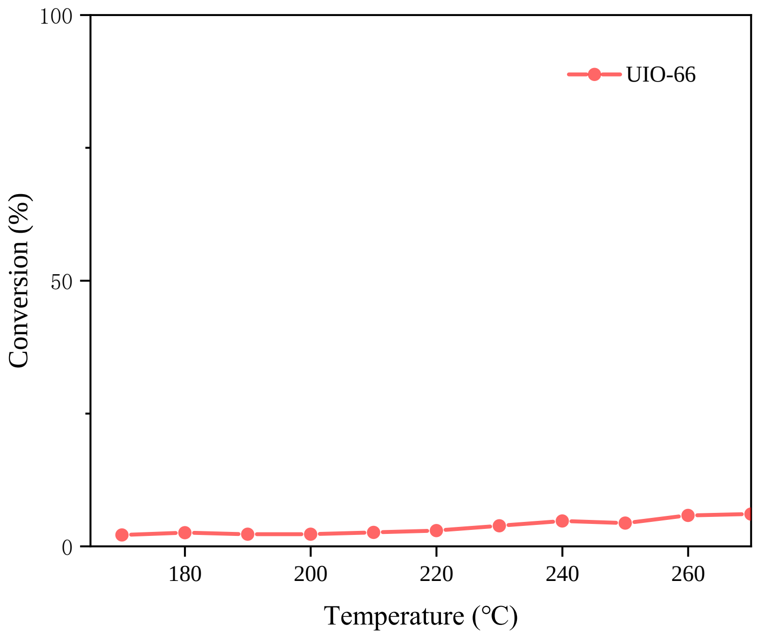

Figure S3. Toluene conversion of UIO-66.

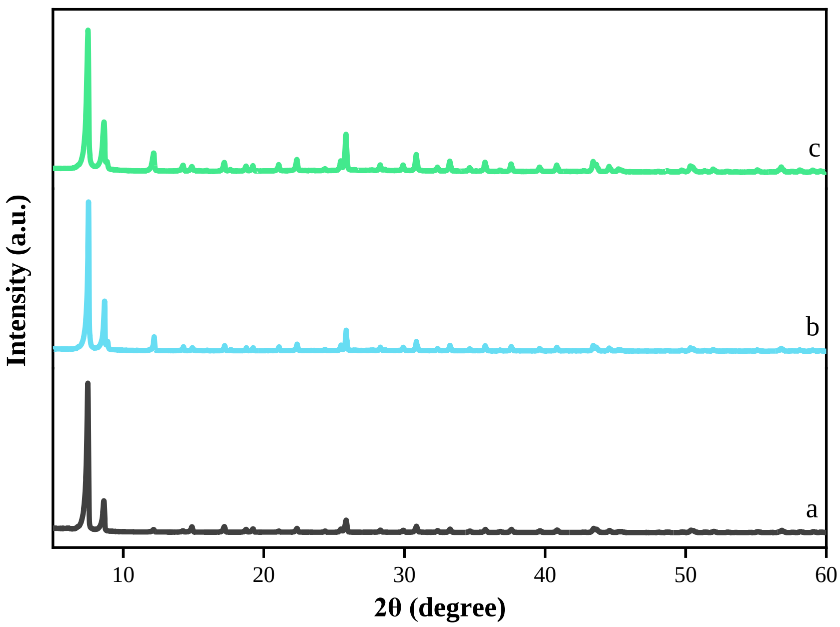

Figure S4. XRD pattern of the sample before and after the reaction, (**a**) UIO-66 (**b**) 0.2% Pd@U before reaction (**c**) 0.2%Pd@U after reaction.

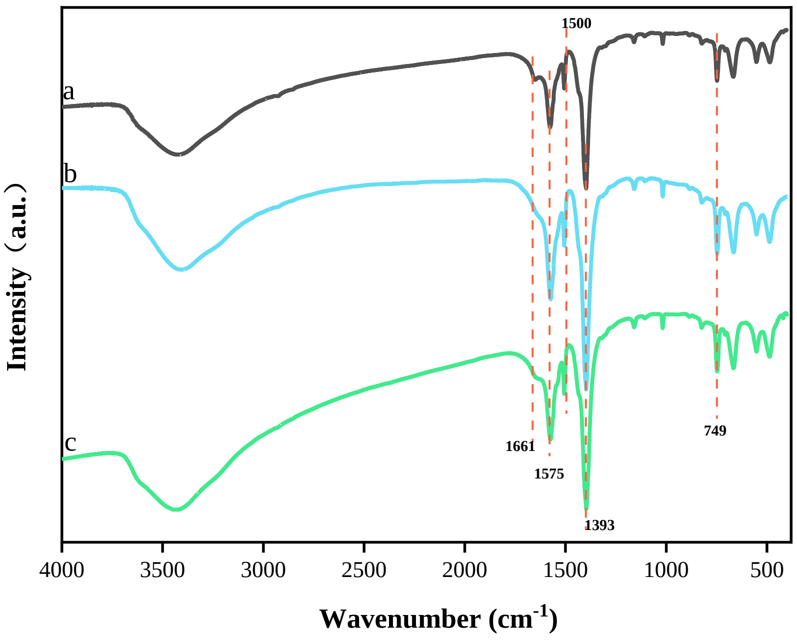

Figure 5. FTIR spectra of (**a**) UIO-66, (**b**) 0.2% Pd@U before reaction and (**c**) 0.2% Pd@U after reaction.

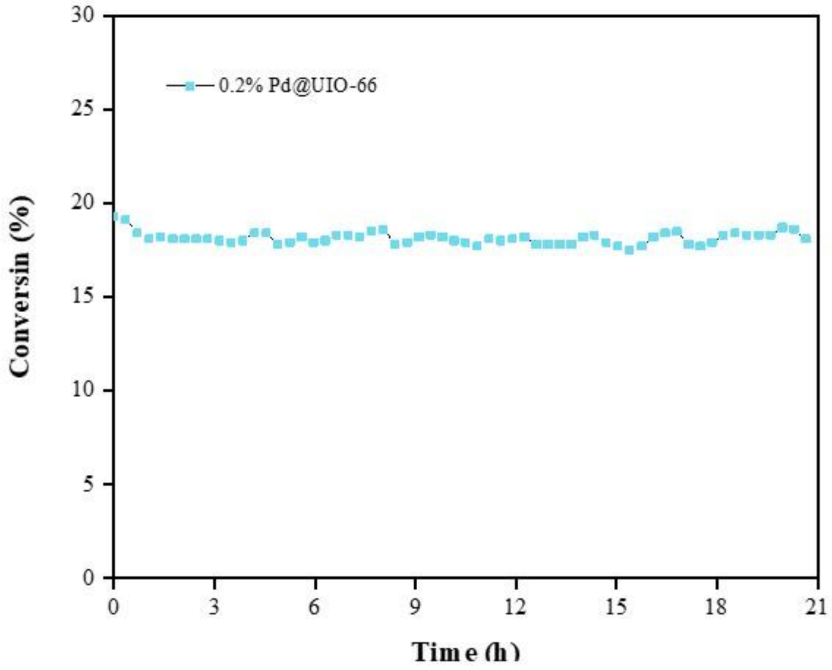

Figure S6. Long-term activity test for 0.2% Pd@U.

Table S1. The Pd content and BET of all sample.

Sample	Pd（wt %[a]）	SBET（$m^2\ g^{-1}$）	Pore vol（$cm^3\ g^{-1}$）	Pore size (Å)
0.1% Pd@UIO-66	0.1	1044.91	0.553	21.18
0.2% Pd@UIO-66	0.2	1012.94	0.525	21.14
1% Pd@UIO-66	1	287.96	0.150	21.64
2% Pd@UIO-66	2	278.17	0.146	21.53
UIO-66	—	1067.45	0.555	21.80

a) Pd content was detected by ICP method.

Table S2. XPS data of the sample.

Sample	Pd^0 (%atom)[a]	Pd^0 BE(eV)	OAds (%atom)[b]	OAds BE(eV)	OLatt (%atom)[c]	OLatt BE(eV)	Zr BE（eV）
0.1%Pd@UIO-66	88.33	333.38	86.55	531.85	13.45	530.10	182.66
0.2%Pd@UIO-66	91.91	333.46	87.32	531.90	12.68	530.18	182.78
1%Pd@UIO-66	90.00	333.57	90.12	531.88	9.88	530.15	182.83
2%Pd@UIO-66	89.51	333.53	92.89	531.91	7.11	530.13	182.94

a) The ratio of surface Pd^0 to (Pd^0 + Pd^{2+}).b) Surface adsorbed oxygen.c) Lattice oxygen.

Table S3. Catalytic Activities and apparent activation energies (Ea) of the four samples.

Sample	catalytic activity (°C)		Ea(kJ mol^{-1})
	T_{50}	T_{90}	
0.1%Pd@UIO-66	232	238	76.88
0.2%Pd@UIO-66	210	217	69.84
1%Pd@UIO-66	236	246	85.18
2%Pd@UIO-66	238	249	87.56
